# Case Report: Endovascular repair for true lumen collapse after frozen elephant trunk for type A aortic dissection

**DOI:** 10.3389/fcvm.2026.1961251

**Published:** 2026-10-12

**Authors:** Xiaoping Fan, Kunyang He, Wenliu Xu, Zengxiao Zou, Naijimuding Abudurexiti, Ge Wang, Chaojie Wang

**Affiliations:** Department of Cardiovascular Surgery, Guangdong Provincial Hospital of Chinese Medicine, The Second Affiliated Hospital of Guangzhou University of Chinese Medicine, Guangzhou, China

**Keywords:** case report, endovascular repair, frozen elephant trunk, malperfusion, true lumen collapse, type a aortic dissection

## Abstract

**Introduction:**

Frozen elephant trunk (FET) was applied in the treatment of type A aortic dissection, enabling one-stage repair of the aortic arch and proximal descending aorta while promoting true lumen expansion. However, inadvertent FET deployment into the false lumen is a rare but catastrophic complication that may cause true lumen collapse and life-threatening visceral malperfusion, with limited reports on its endovascular management.

**Case presentation:**

Here, we present the case of a 44-year-old female who underwent Bentall procedure, total arch replacement, and FET implantation for acute type A aortic dissection at a local hospital. Postoperatively, she developed visceral malperfusion manifesting as abdominal distension, hepatic and renal dysfunction, and elevated lactate levels. Computed tomography angiography revealed that the FET stent had been inadvertently deployed into the false lumen, resulting in true lumen collapse. Emergency thoracic endovascular aortic repair was performed, establishing a “FET-false lumen-true lumen” pathway with an additional stent graft to redirect aortic flow into the true lumen. Computational fluid dynamics analysis showed that the postoperative hemodynamics have been significantly improved. The patient's organ function normalized, and she was discharged two weeks later, remaining asymptomatic at 8-month follow-up.

**Conclusion:**

Endovascular reintervention with additional stent grafting to establish a flow-redirection pathway is a feasible and effective strategy for true lumen collapse following inadvertent FET deployment into the false lumen. Prompt recognition, timely imaging confirmation, and immediate intervention are paramount to mitigating the risks associated with this serious complication.

## Introduction

Frozen elephant trunk (FET) was applied in the treatment of type A aortic dissection, enabling one-stage repair of the aortic arch and proximal descending aorta while facilitating true lumen expansion and false lumen thrombosis (1, 2). However, inadvertent FET deployment into the false lumen is a rare but catastrophic complication that may cause true lumen collapse and life-threatening malperfusion of visceral organs, spinal cord, or lower extremities (3, 4). Prompt recognition and urgent reintervention are therefore critical. Endovascular salvage strategies, including flap fenestration and additional stent grafting to redirect flow into the true lumen, have shown efficacy, yet reported experiences are limited.

Herein, we report a case of true lumen collapse after inadvertent FET deployment into the false lumen, managed with endovascular reintervention and evaluated by CFD analysis of pre- and post-procedural hemodynamics. The Guangdong Provincial Hospital of Chinese Medicine Ethics Committee approved this retrospective review (ZE2022-303-01; approved October 11, 2022) and the patient provided informed written consent for the publication of the data.

## Case report

A 44-year-old female underwent Bentall procedure, total arch replacement combined with FET (Cronus®, Microport Medical Corp., Shanghai, China) implantation for acute type A aortic dissection at a local hospital. Postoperatively, she developed abdominal distension, impaired liver and renal function along with elevated lactate levels, which failed to improve with medical management. Computed tomography angiography (CTA) was subsequently arranged, revealing that the FET stent had been inadvertently deployed into the false lumen, resulting in collapse of the true lumen (Figure 1). As the visceral branches, including the celiac artery, superior mesenteric artery, and both renal arteries, originated from the compressed true lumen, visceral malperfusion ensued.

**Figure 1:**
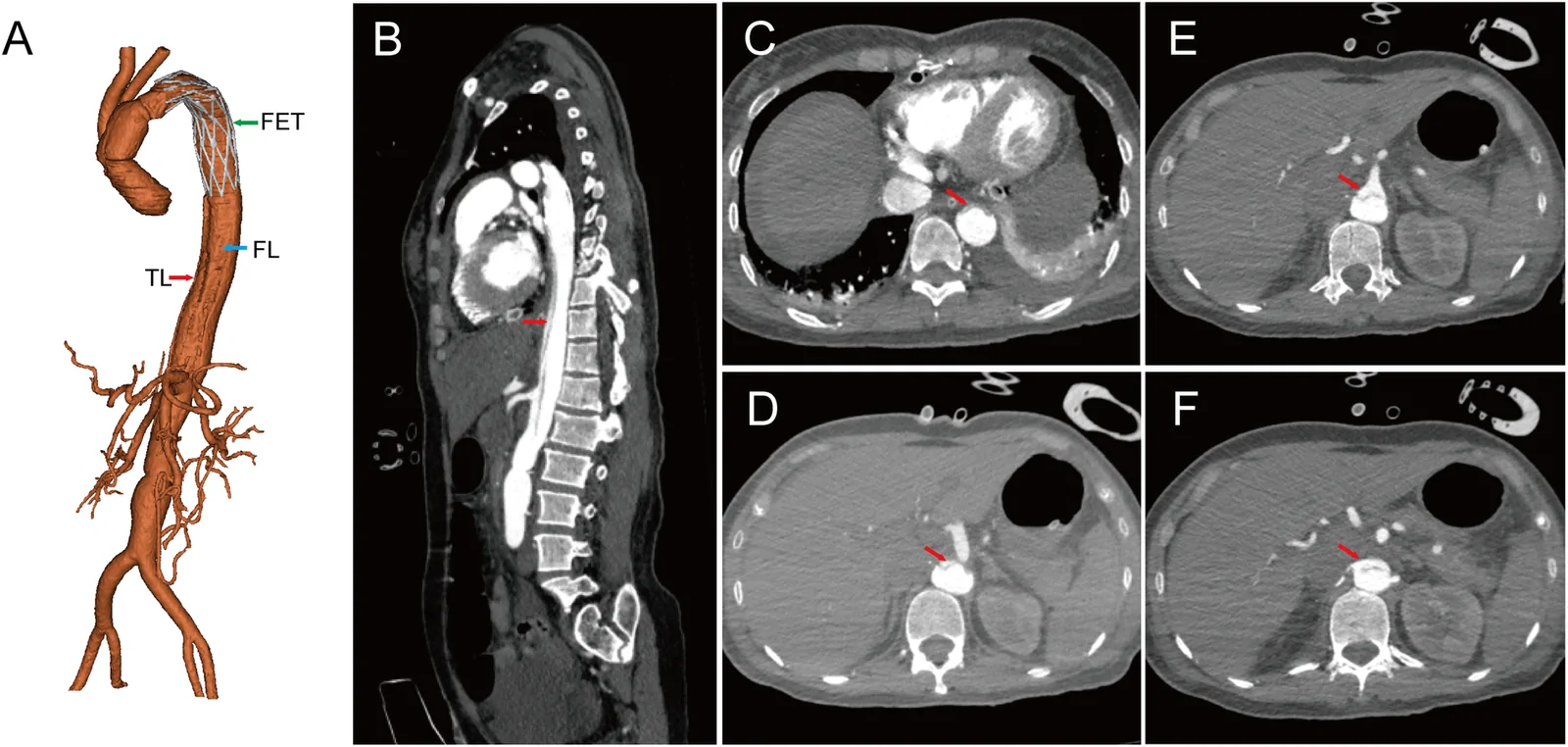
Computed tomography angiography before intervention. **(A)** Three-dimensional reconstruction of the revealed that the FET was inadvertently deployed into the false lumen resulting in true lumen collapse. **(B–F)** Computed tomography demonstrated true lumen collapse, with the visceral branches originating from the compressed true lumen, leading to visceral malperfusion. TL, true lumen (red arrow); FL, false lumen; FET, frozen elephant trunk.

Computational fluid dynamics (CFD) was utilized to analyze the hemodynamics of aortic dissection with true lumen collapse. (Figure 2). The results indicate that the pressure within the false lumen of the descending aorta at the end of systole is relatively high, whereas the pressures in the true lumen and the visceral branch vessels are comparatively low. The wall shear stress of the true lumen is relatively diminished. Blood flow velocity is higher in the false lumen, while it is lower in the true lumen and the visceral branch vessels. Throughout the entire cardiac cycle, the flow rates in the true lumen and the visceral branches remain low, in contrast to the higher flow rate observed in the false lumen.

**Figure 2:**
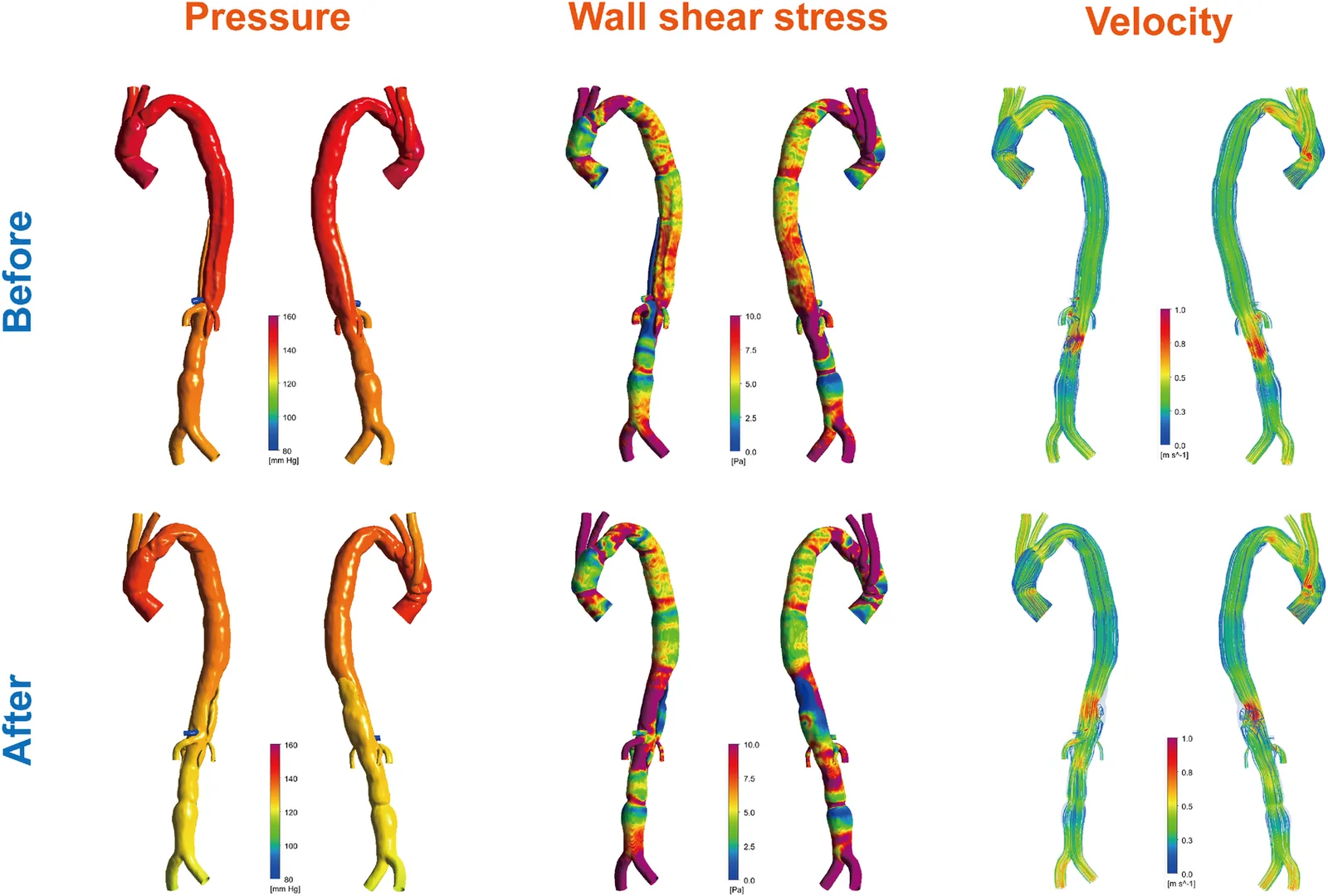
Computational fluid dynamics. At the end-systole, pressure in the acceding aorta and the false lumen of the descending aorta decreased significantly after TEVAR. Wall shear stress increased in the true lumen and decreased in the false lumen. Blood flow velocity increased significantly in the true lumen and the branch vessels, while decreased in the false lumen.

After being transferred to our hospital, emergency thoracic endovascular aortic repair (TEVAR) was performed (Figure 3 and Supplementary Video S1). Initially, angiography of the descending aortic false lumen was conducted (Supplementary Video S2), showing direct blood flow from the FET stent into the false lumen and delayed opacification of the visceral branches. This was followed by true lumen angiography (Supplementary Video S3), which demonstrated a narrowed true lumen without proximal communication to the false lumen; the visceral branches arose from this compressed segment. Through the compressed true lumen, the intimal tear close to the FET stent was superselectively cannulated to access the false lumen. Then, a guidewire was advanced retrograde into the FET stent, establishing a “FET-false lumen-true lumen” pathway. A subsequent thoracic endovascular stent (GORE TAG Thoracic Endoprosthesis, W.L. Gore & Associates, Inc., Arizona, USA) was deployed to redirect aortic flow into the true lumen, thereby restoring perfusion to the visceral branches. The aortic angiography confirmed flow through the FET and the newly placed stent into the true lumen, with earlier and improved opacification of the visceral branches compared to the preoperative state (Supplementary Video S4).

**Figure 3:**
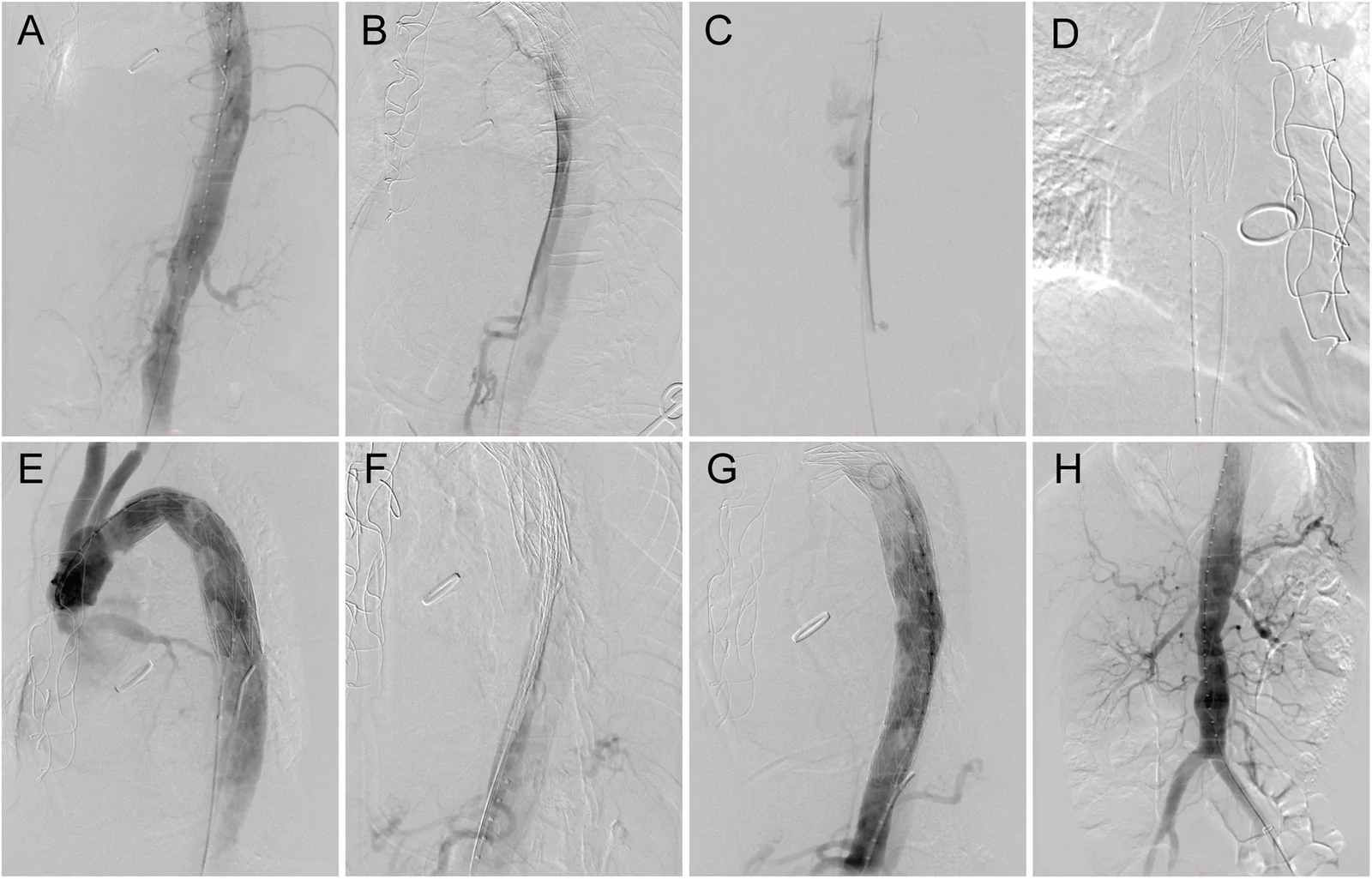
Intraoperative procedures. **(A)** Angiography of the descending aortic showed direct blood flow from the FET stent into the false lumen. **(B)** True lumen angiography demonstrated a narrowed true lumen and the visceral branches arose from this compressed segment. **(C)** Small intimal tears were identified distal to the stent graft. **(D)** Through the compressed true lumen, the tear close to the FET was superselectively cannulated to access the false lumen. **(E)** A guidewire was then advanced retrograde into the FET stent, establishing a “FET-false lumen-true lumen” pathway. **(F)** A thoracic endovascular stent was deployed to redirect aortic flow into the true lumen. **(G)** The aortic angiography confirmed flow through the FET stent and the newly placed stent into the true lumen. **(H)** The aortic angiography confirmed that the visceral branches were well perfused.

Following TEVAR, the patient's abdominal distension, liver and renal function normalized, and lactate levels decreased. No spinal cord ischemia or stent migration occurred. Follow-up CTA demonstrated significant expansion of the true lumen and shrinkage of the false lumen in the descending aorta (Figure 4). The patient was discharged two weeks later and remained asymptomatic at the 8-month follow-up.

**Figure 4:**
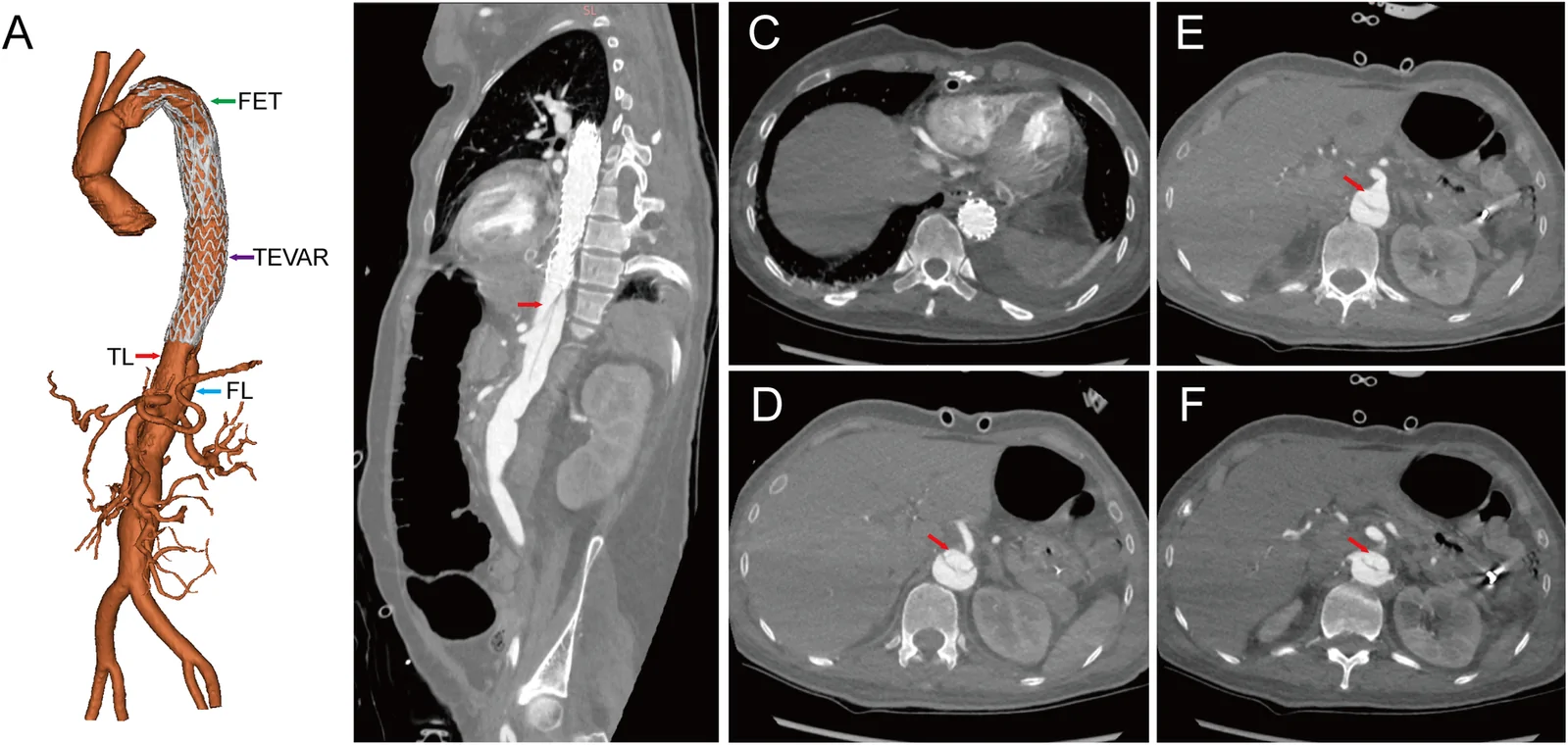
Computed tomography angiography after intervention. **(A)** The three-dimensional reconstruction of the aorta after the intervention showed that the stent had returned to the true lumen. **(B–F)** Follow-up CTA demonstrated significant expansion of the true lumen and shrinkage of the false lumen in the descending aorta. TL, true lumen (red arrow); FL, false lumen; FET, frozen elephant trunk; TEVAR, thoracic endovascular aortic repair.

CFD analysis was performed to evaluate hemodynamic changes before and after thoracic endovascular stent graft placement (Figure 2). The results indicated that, at the end-systole, pressure in the acceding aorta and the false lumen of the descending aorta decreased significantly after TEVAR. Wall shear stress increased in the true lumen and decreased in the false lumen. Blood flow velocity increased significantly in the true lumen and the branch vessels, while decreased in the false lumen. Over the cardiac cycle, flow in the true lumen and the visceral branches increased, whereas flow in the false lumen diminished.

## CFD methods

Patient-specific geometries were reconstructed from CTA data using the image analysis software Mimics 21.0 (Materialise, Belgium). Segmentation was performed using a semi-automatic threshold-based method combined with region growing. Following reconstruction, manual corrections were applied to improve the reliability and precision of the segmentation. Unstructured tetrahedral meshes were then generated using ICEM (ANSYS, Canonsburg, Pennsylvania). CFD simulations were conducted using CFX (ANSYS, Canonsburg, Pennsylvania). The velocity and pressure fields were obtained by solving the Navier-Stokes equations under prescribed boundary conditions. For the outlet boundary conditions, a three-element Windkessel model was applied at all outlets. Post-processing and visualization of the simulation results were carried out using CFD-Post (ANSYS, Canonsburg, Pennsylvania).

## Comment

In the management of acute type A aortic dissection, the inadvertent deployment of FET into the false lumen resulting in true lumen collapse is a rare but devastating complication (3, 4). This maldeployment can lead to malperfusion of visceral organs, the spinal cord, or the lower extremities, and is associated with high mortality (5, 6). Therefore, rapid recognition and timely, effective intervention are crucial to mitigating the adverse outcomes associated with incorrect stent placement.

Prompt identification of stent malposition is critical for patient salvage. Intraoperatively, routine monitoring of the pressure gradient between upper and lower limb arteries should be performed. Postoperatively, signs of visceral malperfusion, characterized by acute liver and kidney dysfunction, coagulopathy, elevated lactate and myoglobin levels, reduced urine output, and metabolic acidosis, must be closely monitored. If stent malposition is suspected, immediate confirmation with aortic CTA or angiography is mandatory. Once confirmed, emergency intervention is required to address the malperfusion. This may involve additional stent grafting to redirect flow into the true lumen, either through existing intimal tears or via fenestration created between the FET and the celiac artery, effectively establishing a “FET-false lumen-true lumen” pathway (7, 8).

The application of CFD provides valuable visual insights into hemodynamic parameters within the aorta. This modality enables the non-invasive and intuitive quantification of key hemodynamic parameters, including pressure, wall shear stress, flow velocity, flow direction, and volume (9, 10). Through CFD analysis, we can quantitatively assess preoperative and postoperative hemodynamic changes, offering a scientific basis and potential for predicting the outcomes of interventions before they are performed (11, 12).

In summary, when malposition of FET stent is suspected, immediate imaging with CTA or angiography is mandatory. A comprehensive assessment of the anatomy and hemodynamic status is essential to formulate an appropriate intervention strategy. Rapid recognition and timely, effective intervention are paramount to mitigating the risks associated with this serious complication.

## Data Availability

The raw data supporting the conclusions of this article will be made available by the authors, without undue reservation.

## Supplementary material

Supplementary Video S1 Emergency endovascular repair procedure for true lumen collapsed.

Supplementary Video S2 Angiography of the false lumen before intervention.

Supplementary Video S3 Angiography of the true lumen before intervention.

Supplementary Video S4 Angiography of the descending aorta after intervention.
